# Long-Distance Repression by Human Silencers: Chromatin Interactions and Phase Separation in Silencers

**DOI:** 10.3390/cells11091560

**Published:** 2022-05-05

**Authors:** Ying Zhang, Yi Xiang See, Vinay Tergaonkar, Melissa Jane Fullwood

**Affiliations:** 1 Cancer Science Institute of Singapore, National University of Singapore, 14 Medical Drive, Singapore 117599, Singapore; csiyz@nus.edu.sg (Y.Z.); yixiang.see@ntu.edu.sg (Y.X.S.); 2 NUS Centre for Cancer Research, Yong Loo Lin School of Medicine, National University of Singapore, 14 Medical Drive, Singapore 117599, Singapore; 3 School of Biological Sciences, Nanyang Technological University, 60 Nanyang Drive, Singapore 637551, Singapore; 4 Institute of Molecular and Cell Biology, Agency for Science, Technology and Research (A*STAR), 61 Biopolis Drive, Proteos, Singapore 138673, Singapore; vinayt@imcb.a-star.edu.sg; 5 Department of Pathology, Yong Loo Lin School of Medicine, National University of Singapore (NUS), Singapore 117597, Singapore

**Keywords:** silencers, chromatin interactions, phase separation

## Abstract

Three-dimensional genome organization represents an additional layer in the epigenetic regulation of gene expression. Active transcription controlled by enhancers or super-enhancers has been extensively studied. Enhancers or super-enhancers can recruit activators or co-activators to activate target gene expression through long-range chromatin interactions. Chromatin interactions and phase separation play important roles in terms of enhancer or super-enhancer functioning. Silencers are another major type of cis-regulatory element that can mediate gene regulation by turning off or reducing gene expression. However, compared to active transcription, silencer studies are still in their infancy. This review covers the current knowledge of human silencers, especially the roles of chromatin interactions and phase separation in silencers. This review also proposes future directions for human silencer studies.

## 1. Introduction

Humans are complex multicellular organisms made up of 30–40 trillion cells, all containing the same DNA sequences [1]. Yet, different cell types express distinct sets of genes to perform vastly different functions within the human body. *Cis*-regulatory elements (CREs) are non-coding sequences that play an important role in regulating these cell type-specific transcriptional programs that allow for heterogeneous gene expression from a common DNA template [2,3]. CREs are enriched for transcription factor binding sites that activate or repress the transcription of associated genes [4]. In this review, we cover the present knowledge about repressive CREs known as silencers, including how three-dimensional (3D) genome organization participates in repressing gene expression.

Extensive research has been done on two classes of activating CREs: promoters and enhancers. Promoters are located proximal to transcription start sites and contain binding sites for RNA polymerase II and other general transcription factors that initiate transcription [5,6,7]. Enhancers are similarly enriched in binding sites for activating transcription factors and cofactors, but are located linearly distant from gene promoters in either direction [8]. Chromatin looping brings enhancers and their target promoters close together in three-dimensional space, allowing regulatory signals to be transmitted from enhancers to promoters across the long linear distances [9,10,11]. Active promoters and enhancers are respectively marked by trimethylation and monomethylation of lysine 4 of histone H3 (H3K4me3 and H3K4me1) [12], and both are marked by acetylation of lysine 27 of histone H3 (H3K27ac) [13]. Multiple enhancers can cooperate additively or synergistically to regulate gene expression [8].

Just as activating CREs are required to activate selected genes for cell type-specific functions, so too are repressive CREs required to curb the expression of genes that need to be switched off. Although activating CREs are well characterized, repressive CREs remain poorly understood. Silencers are one type of repressive CRE, broadly defined as sequence-specific CREs that switch off or reduce the expression of their target genes. Unfortunately, very few human silencers have been identified and characterized. In Table 1, we show a partial list of experimentally validated human silencers.

Silencers can be classified into proximal and distal silencers depending on their position relative to the gene promoter. Promoter-proximal silencers are usually position dependent and contain binding sites for repressor proteins that can switch off gene expression by inhibiting transcriptional machinery binding or function [14]. Promoter-distal silencers are usually position and orientation independent and can loop to target promoters to repress gene expression, functioning as the repressive analogue to enhancers [14,15].

**Table 1:** Validated examples of human silencers.

Silencer Position	Target Gene	Proximal or Distal	Reference
Promoter	synapsin I (*SYN1*)	proximal	[16]
Promoter	interferon gamma (*IFNG*)	proximal	[17]
Promoter	platelet-derived growth factor subunit A (*PDGFA*)	proximal	[18]
Intron	human CD4 molecule (*CD4*)	proximal	[19,20]
Exon	chimerin 1 (*CHN1*)	proximal	[21]
Intron	collagen type IV alpha 2 chain (*COL4A2*)	proximal	[22]
Promoter	thyroid-stimulating hormone subunit beta (*TSHB*)	proximal	[23]
Promoter	serpin family B member 2 (*SERPINB2*)	proximal	[24]
Promoter	glutathione S-transferase pi 1 (*GSTP1*)	proximal	[25]
Intron	apolipoprotein A2 (*APOA2*)	proximal	[26]
Intron and UTR	methyl CpG-binding protein 2 (*MECP2*)	proximal	[27]
15 putative silencers	unknown (silencing activity characterized using functional assays)	distal	[28]
Intergenic	cyclinD1 (*CCND1*)	distal	[29]
Intron	Rho GTPase-activating protein 6 (*ARHGAP6*)	proximal	[30]
5 H3K27me3-DNase hypersensitive sites	unknown (silencing activity characterized using functional assays)	distal	[31]
Methylation-rich region	human fibroblast growth factor 18 (*FGF18*)	distal	[32]
Methylation-rich region	human insulin like growth factor 2 (*IGF2*)	distal	[32]

## 2. Genome-Wide Identification of Silencers

**Figure 1:**
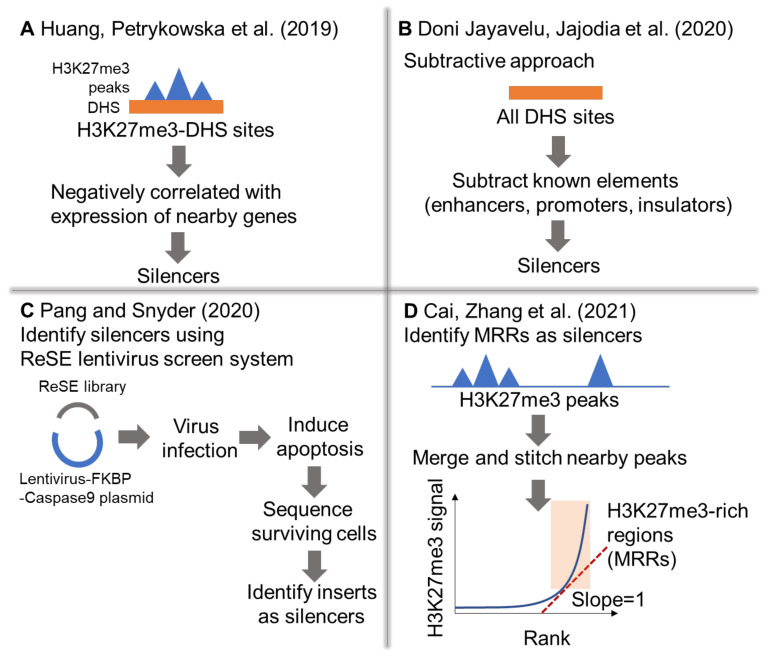
Different identification methods of human silencers. (**A**) H3K27me3-DHS sites that were negatively correlated with expression of nearby genes in different cell lines were termed silencers. DHS—DNase I hypersensitive sites. (**B**) Identifying silencers using the subtractive approach. (**C**) Identifying human silencers by high-throughput functional screen via measuring the repressive ability of silencer elements (ReSE). (**D**) Identifying H3K27me3-rich regions (MRRs) as silencers.

With more and more individual silencer examples, systematic methods identifying human silencers have been proposed. Huang et al. [31] identified silencers in the human genome by selecting for heterochromatic loci that were accessible to transcription regulation machinery, using H3K27me3 ChIP-seq peaks and DNase I hypersensitive sites (DHS) (Figure 1A). They assigned these H3K27me3-DHS sites to the nearest genes and explored their correlation with expression across multiple cell lines. H3K27me3-DHS sites that were negatively correlated with gene expression in different cell lines were termed as putative silencers. Five out of ten putative silencers showed decreased luciferase reporter gene activity, demonstrating the prediction power of this method. Moreover, transcriptional repressors such as CCCTC-binding factor (CTCF), SMAD family member 4 (SMAD4), and snail family transcriptional repressor 3 (SNAI3) were enriched at these putative silencers.

Doni Jayavelu et al. [33] adopted a subtractive approach to identifying putative silencers (Figure 1B). They first used DNase I hypersensitive sites to denote open chromatin regions in the genome. Enhancers, promoters, insulators, and active transcription start sites were then subtracted from the open chromatin regions, and the remaining open chromatin regions were termed as putative silencers. They performed validation on 7430 putative silencer elements via massively parallel reporter assays (MPRA) using self-transcribing active regulatory region sequencing (STARR-seq) in K562 cells. The validation results showed that the putative silencer elements in K562 had comparable transcription activities compared with randomly selected regions, demonstrating limited predictive power. Only around half of the putative silencer elements had lower transcription activity compared to the median of randomly selected regions. Nevertheless, they still found that these putative silencer elements enriched for known repressors such as RE1 silencing transcription factor (REST), YY1, zinc finger and BTB domain-containing 33 (ZBTB33), SUZ12, and EZH2.

In contrast to the above methods, Pang and Snyder [34] systematically identified silencers through a high-throughput functional screen rather than predicting using epigenetic marks or chromatin interactions (Figure 1C). This method measured the repressive ability of silencer elements (ReSE) by screening for genomic fragments that repress the transcription of an apoptosis-inducing protein (a modified caspase 9 fused to an FK506-binding protein (FKBP-Casp9)). If the inserted fragments have silencer activity, it will repress the transcription of the FKBP-Casp9 gene in the cells, thus preventing apoptosis. By expanding surviving cells and sequencing the inserts within these cells, silencer regions can be identified. Overall, they identified 2664 putative silencer regions in K562 cells, and three silencer regions located in the intron regions of *HRH1*, *SYNE2*, and *CDH23* were validated via CRISPR/Cas9 deletion.

Our group followed a similar approach to Huang et al. [31] and identified H3K27me3-rich regions (MRRs) as putative silencers based on H3K27me3 ChIP-seq signal (Figure 1D) [32]. H3K27me3 peaks in close proximity were clustered together, and these clusters were ranked by their H3K27me3 enrichment. Similar to super-enhancer identification, clusters with the highest H3K27me3 ChIP-seq signals were termed as H3K27me3-rich regions (MRRs). A total of 974 MRRs were identified in K562 cells, and 2 MRRs were validated for their silencing functions via CRISPR/Cas9 deletion.

Although various systematic methods have been proposed [31,32,33,34,35], there is no consensus yet in terms of how to identify human silencers. Each of these methods identifies different genomic regions as human silencers; however, validation of these silencers has not been sufficient. MRRs from Cai et al. [32] were compared to ReSE silencer elements from Pang and Snyder [34] in the K562 cell line; only 10.66% of putative silencers overlapped between both sets, although the overlap was still significantly higher than with random control. This suggests that there are indeed some common human silencers which can be called upon by the cell for different functions; however, unique human silencers found by particular methods also exist, raising the possibility that there may be different classes of human silencers. Therefore, establishing a gold standard for the identification of human silencers would be an important future direction for this field.

## 3. Silencers Interfere with Binding of Activators and Transcriptional Machinery

**Figure 2:**
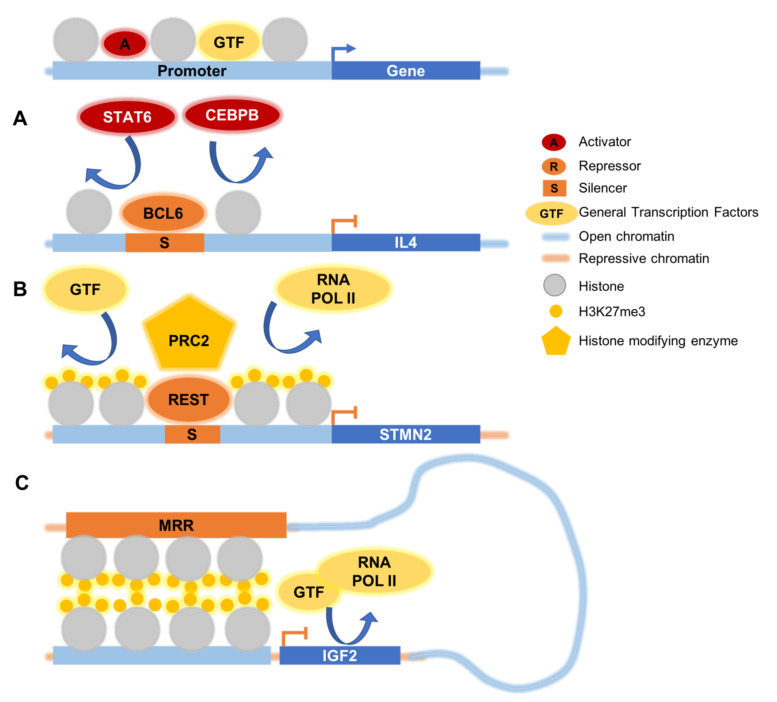
Mechanisms for silencer repression. Silencers can repress gene expression in two ways: (**A**) One way is to compete with activators or general transcription factors (GTF) for binding sites. For example, BCL6 competes with STAT6 and CEBPB for binding at the *IL4* promoter to prevent transcription [36]. (**B**) Another way is to generate a repressive chromatin environment, for example by methylating the histones at the gene promoter, thereby preventing the binding of activators and transcriptional machinery. For example, the REST complex binds at the promoter of *STMN2* [37], recruiting the PRC2 complex and depositing H3K27me3. (**C**) Silencers can interact with linearly distant gene promoters through chromatin looping, to perform their repressive functions. The *IGF2* promoter interacts with a distal H3K27me3-rich region (MRR) and forms a repressive chromatin structure [32]. CRISPR/Cas9 excision of the MRR increases *IGF2* transcription.

In general, silencers function in two ways: First, repressors may bind at silencers to block binding sites for activators and transcription machinery (Figure 2A). For example, transcriptional repressor BCL6 can repress interleukin 4 (*IL4*) gene expression in B cells by competing with signal transducer and activator of transcription 6 (STAT6) and CCAAT enhancer binding protein beta (CEBPB) for promoter binding [36].

Second, silencers may prevent activators and/or GTFs from accessing promoters by establishing a repressive chromatin structure through the recruitment of histone modifiers or chromatin stabilizing factors (Figure 2B). In particular, the repressive remodeling of chromatin structure frequently involves histone methylation and deacetylation.

## 4. Histone Methylation at Silencers

Histone proteins are decorated by an array of post-translational modifications, including methylation and acetylation, that regulate chromatin accessibility and transcription factor binding [38]. Methylation of different histone lysine residues are recognized by specific ‘reader’ proteins that can activate or repress transcription [39]. In particular, repressed constitutive (stable) and facultative (dynamic) heterochromatin domains are associated with H3K9me3 and H3K27me3. Although it is not completely clear how these histone methylation modifications direct transcription silencing, recent research suggests that they interact with H1 linker histones to compact chromatin and repress transcription [40,41].

Polycomb group (PcG) proteins form Polycomb repressive complexes (PRC) that deposit H3K27me3 marks on facultative heterochromatin [42]. Global screens for human silencers indicate that PRC complexes are enriched at these CREs [32,33,34]. EZH2, which is the enzymatic subunit of PRC2 that catalyzes H3K27 tri-methylation, was found to be enriched at putative human silencers identified by Doni Jayavelu et al. [33] and Pang and Snyder [34]. Our recent work also uncovered exquisite enrichment of EZH2 at putative silencers, with exceptionally high enrichment of H3K27me3 (termed as methylation-rich regions or MRRs) compared to typical H3K27me3 peaks [32].

Individual silencers have been shown to be enriched for repressors that recruit PcG proteins for their repressive function. The repressor element 1 (RE-1) is a silencer first identified in humans upstream of the stathmin 2 gene (*STMN2*) [37], and subsequently identified upstream of the synapsin I gene (*SYN1*) [16]. RE-1 was shown to be occupied by a repressor protein (RE-1 silencing transcription factor REST) in non-neuronal cell lines, but the repressor was absent in neuronal cell lines, demonstrating cell-type specificity in function [16,37]. Using a luciferase reporter assay, RE-1 was shown to silence reporter gene expression, and mutation or deletion of this silencer induced upregulation of the reporter gene [16].

The multifunctional transcriptional factor YY1 can repress gene expression in some specific conditions by recruiting PRC2 [43,44,45], creating a repressive chromatin structure that prevents activator binding. Together, these pieces of evidence suggest that PcG proteins are involved in a class of silencer function.

## 5. Histone Deacetylation at Silencers

Besides histone methylation, chromatin compaction is also correlated with histone deacetylation. Acetyl groups reduce the positive charges on histones, which decreases electrostatic interactions with chromatin, thereby relaxing and opening the chromatin structure for transcription factor binding [38]. Hence, the removal of these acetyl groups by histone deacetylases creates a more compact chromatin structure that inhibits the binding of activators and transcriptional machinery. Notably, many repressors recruit histone deacetylases HDAC1 and HDAC2, which are found in multiple histone deacetylation complexes such as the SIN3A-HDAC complex, the nucleosome remodeling and deacetylase complex (NuRD), and the CoREST complex structure [38].

For example, SP1 and SP3 have been shown to function as repressors by recruiting SIN3A-HDAC complexes, inhibiting the expression of genes such as luteinizing hormone receptor (*LHCGR*) [46,47], telomerase reverse transcriptase (*TERT*) [48], interleukin 1 alpha (*IL1A*) [49], cyclin dependent kinase inhibitor 1A (*CDKN1A*) [50], and endothelial PAS domain protein 1 (*EPAS1*) [51]. The RUNX family transcription factor 1 (RUNX1) binds at a silencer in the first intron of the *CD4* gene [52], recruiting SIN3A/HDAC complexes to repress transcription [53]. Notably, this silencer exhibited both orientation and position independent negative regulatory activity [20,54], and demonstrated cell type-specificity in function, repressing reporter gene expression selectively in CD4^-^/CD8^-^ double-negative and CD4^-^/CD8^+^ single-positive T cells but not in CD4^+^/CD8^-^ single-positive T cells [20]. POU2F1 acts as a repressor when binding to an A+T rich silencer motif at promoters by recruiting the NuRD complex [55], inhibiting transcription of genes such as *TSHB* [23], *CYP1A1* [56], *POU1F1* [57], and *NOS2* [58].

## 6. Distal Silencers Loop to Promoters to Inhibit Gene Expression

Just as enhancers or super-enhancers form chromatin interactions with distant gene promoters to activate transcription [59,60,61], so too can distal silencers loop to distant target genes to repress transcription. In particular, H3K27me3-marked domains connect together via a network of chromatin interactions to create a compact and inaccessible structure.

Looping silencers have been well documented in Drosophila [62]. The Drosophila Snail (Sna) protein is a well-known repressor of non-mesodermal genes in the developing mesoderm [63]. Gisselbrecht et al. [64] showed that a subgroup of silencers was significantly enriched for chromatin interactions with transcription start sites, and H3K27me3 signals at these silencers were anti-correlated with the expression of interacting genes. This suggests that some silencers work through recruiting repressors to mediate silencing activity. Chromatin loops also connect H3K27me3-marked Polycomb repressive elements (PREs) and promoters, repressing gene expression [62,65,66].

Chromatin loops similarly connect H3K27me3-marked domains in mice. Using Chromatin Interactions Analysis with Paired-End Tag sequencing (ChIA-PET), Ngan et al. [35] enriched for chromatin interactions in mouse embryonic stem cells that involve PRC2, which deposits H3K27me3 modifications. They revealed that genes interacting with PRC2-bound loci tend to be lowly expressed, demonstrating long-range repression via chromatin looping. CRISPR/Cas9-mediated deletion of 21 PRC2-bound loci reactivated expression of interacting genes, adding to the evidence that chromatin interactions play important roles in silencers function. In vivo deletion of 6 PRC2-bound silencers caused pleiotropic developmental defects in mice, highlighting the importance of silencers in development.

In humans, few examples of looping silencers have been identified. Hi-C and ChIA-PET analysis in K562 cells identified a silencer proximal to the *ABCC2* gene that connected to *CPN1* through chromatin looping [34]. CRISPR/Cas9-mediated deletion of this silencer significantly upregulated the expression of both the proximal *ABCC2* and the distal *CPN1*, validating the long-range repressive function of this silencer. Apart from the validated *ABCC2* looping silencer, the same study also intersected promoter capture Hi-C data from human primary blood cells and silencers identified from K562 cells, and identified around 4000 silencer–promoter chromatin interactions, suggesting a high prevalence of looping silencers [34]. Another promoter-capture Hi-C analysis revealed chromatin interactions between 12,321 candidate silencers and 17,250 genes in GM12878 cells, while 5907 candidate silencers were found to interact with 8599 genes in CD34^+^ cells [33]; however, their long-range repressive functions were not validated through gene expression analysis.

In our recent work, we identified MRRs with exquisite enrichment of H3K27me3 as putative human silencers and found that MRRs were highly associated with chromatin interactions [32]. MRRs showed extensive looping within clusters and to distant genes. Genes located proximal to MRRs and genes distal to MRRs were both associated with low gene expression to a similar extent, indicating that silencers can function effectively across long distances through chromatin looping. CRISPR/Cas9-mediated excision of an MRR targeting *IGF2* led to the upregulation of both proximal and distal genes, including genes associated with erythroid differentiation and cell adhesion, as well as growth inhibition in xenograft models, suggesting that looping silencers are important in establishing cell identity. Removing the silencer led to decreased H3K27me3 and increased H3K27ac histone modifications at distal loops, opening up the chromatin structure. This suggests that looping silencers function by maintaining a repressive chromatin structure. Taken together, these recent works demonstrate the importance of silencers in maintaining cell identity and show that silencer looping is likely to be a major mechanism of repression.

To the best of our knowledge, apart from our characterization of the *IGF2* looping silencer, there has been no detailed exploration on the mechanism and function of looping silencers. We speculate that repressors may facilitate the formation of chromatin interactions between repressed regions (Figure 2C). Conversely, chromatin interactions may also increase the enrichment of repressors at the gene promoter region, just as how looping enhancers activate target genes by increasing the enrichment of activators (Figure 2C). There is some evidence to support this speculation. For example, YY1 was found to enrich at silencers in multiple cell lines, including K562, H1, GM12878, and HEPG2 cells [32,33]. YY1 has been implicated as a chromatin structural protein in enhancer–promoter chromatin interactions through homodimer formation [67]. Hence, we speculate that YY1 may also function as a repressor by mediating repressive chromatin interactions. Moreover, PRC2 complex subunits EZH2 and SUZ12 were found to enrich at silencers as well [32,66]. PRC2 contributes to chromatin compaction by mediating nucleosome bridging across distances, facilitating chromatin looping [68]. EZH2 inhibition leads to changes in chromatin interactions and increased expression of associated genes [32,69,70], suggesting that PRC2 participates in looping silencer function.

## 7. Phase Separation in Silencing

**Figure 3:**
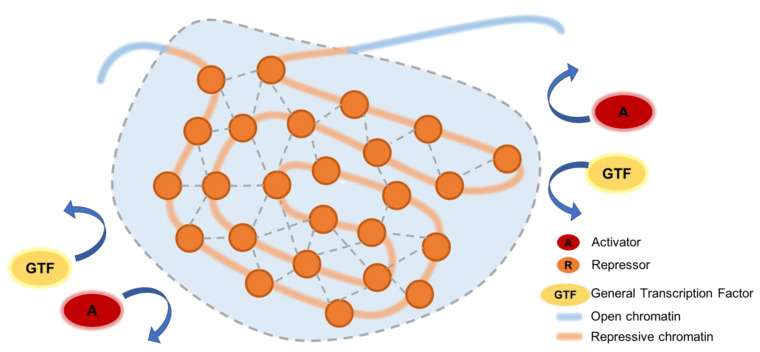
Phase separation in silencing. Repressors bind to heterochromatin domains and form protein–protein interactions with each other, assembling into a phase-separated condensate that selectively incorporates repressive factors and excludes transcription activators.

Recent studies have proposed that transcription activation is mediated by liquid–liquid phase separation (LLPS), concentrating transcription activators within biomolecular condensate compartments at promoter–enhancer interactions [71,72,73,74,75] (reviewed in [76]). These condensates form when their components segregate themselves from other nuclear components because they have higher affinity for each other, leading to liquid–liquid demixing not unlike a mixture of water and oil [77]. LLPS has also been implicated in transcription silencing, notably in constitutive heterochromatin domain formation and facultative heterochromatin-silencing by PcG proteins (Figure 3).

The formation of constitutive heterochromatin domains is mediated by the chromobox 5 protein (CBX5, also known as HP1α), which recognizes and binds to H3K9me2/3 [78,79]. CBX5 oligomerizes and phase-separates in solution in vitro to form liquid-like droplets, compacting bound DNA at the same time [80,81]. CBX5 condensates selectively incorporate heterochromatin-associated factors, including shugoshin 1 (SGO1) [80], methyl-CpG binding protein 2 (MECP2) [82], scaffold attachment factor B (SAFB) [83], and major satellite RNAs [83]. At the same time, these condensates exclude activating transcription factors such as general transcription factor IIB (GTF2B) [84]. The individual interactions between condensate constituents are weak, allowing for dynamic movement within the compartment; however, the accumulation of weak interactions contributes to resistance against mechanical disruptions [85].

Facultative heterochromatin compaction may also be mediated by LLPS of PcG proteins. Canonical Polycomb repressive complex 1 (cPRC1) is assembled from RING1A and RING1B histone ubiquitin ligase proteins, a Polycomb group ring-finger domain protein (PCGF2/4), a Polyhomeotic homologous protein (PHC1/2/3), and a chromobox protein (CBX2/4/6/7/8). CBX7/8 mediates the binding of cPRC1 to H3K27me3-marked regions [86], while CBX2-cPRC1 complexes compact these domains through oligomerization [70,87,88]. Recent papers have shown that CBX2 forms phase-separated condensates on its own [89,90]. These CBX2 condensates can incorporate other components of cPRC1 and H3K27me3-marked chromatin, condensing these domains.

Although preliminary studies have provided strong evidence of heterochromatin condensates in vitro and in vivo, important questions about the formation of these condensates remain unanswered: How do these condensates assemble and disassemble dynamically, especially during cell cycles? Does the formation of these condensates require initial seeding of heterochromatin proteins at specific nucleation sites? The answers to these questions will greatly improve our understanding of chromatin organization and gene regulation.

## 8. Potential Role of Non-Coding RNA (ncRNA) in Silencing

Non-coding RNAs (ncRNA) are key regulators of gene expression. Recently, Long et al. [91] found that PRC2 required ncRNA binding for precise repression of gene expression and cellular differentiation. Specifically, RNase A digestion did not inhibit PRC2 assembly and activity, but disrupted PRC2 chromatin occupancy and localization in human pluripotent stem cells [91]. Additionally, Gavrilov et al. [92] identified a variety of cis-acting ncRNAs enriched at PRC2 binding sites in human embryonic stem cells using RedChIP, including previously known PRC2-associated ncRNAs such as *KCNQ1OT1* [93]. These results highlight the importance of ncRNAs in the process of gene repression. Besides mediating PRC2 recruitment to promoters, ncRNAs also interact with chromatin structural proteins such as CTCF, suggesting a potential role in the formation of CTCF-dependent chromatin loops. However, Barutcu et al. [94] found that TAD boundaries were largely unchanged upon RNase treatment, which makes it unclear what the exact role of ncRNAs during loop and TAD formations is. These results highlight the importance of ncRNAs in the process of gene repression.

Although there are no examples of ncRNA-mediated silencers to date, various ncRNAs have been shown to play a direct role in silencing gene transcription. One of the most well-known examples is X inactive-specific transcript (*XIST*), which orchestrates X chromosome inactivation (XCI) in females [95]. The *XIST* ncRNA triggers a cascade of events, including loss of histone acetylation [96], recruitment of PRCs to deposit H3K27me3 [97], and mediation of chromosome compaction [98]. This results in transcriptional silencing of most genes across one of the two copies of chromosome X in females. *XIST* binds first to chromatin in close spatial proximity to its gene locus, before spreading further to binding sites across the entire chromosome [99], indicating that ncRNAs can exploit the chromatin conformation landscape to effect gene silencing. Other ncRNAs have been implicated in gene repression (for recent reviews, see Guttman and Rinn [100] and Engreitz et al. [99]).

Taken together, these pieces of evidence suggest that ncRNAs may play important roles in silencer function, potentially by recruiting repressors to proximal silencers, or mediating long-range chromatin interactions between distal silencers and target promoters. It would be interesting to dissect the exact role of ncRNAs regarding loop and TAD formation, and repressor recruiting. Specifically, revealing detailed looping silencer examples involving ncRNAs is necessary.

## 9. Silencers in Health and Disease

Human silencers have been shown to repress target genes during development and differentiation processes. For example, a lineage-specific silencer of *CD4* gene was reported to repress *CD4* gene expression during T cell lymphocyte development, which can play roles in cell date determination [20]. The RE1-silencing transcription factor (REST) plays a role in preventing ectopic expression of L1 cell adhesion molecule (*L1CAM*) and other target genes in non-neural tissues during early embryonic development [101]. REST, together with corepressor RCOR1, recruits histone deacetylases, such as the SIN3A- HDAC complexes, and histone methyltransferases, such as EHMT2 and KDM1A, to compact chromatin and reduce accessibility [102].

Genome-wide identification of human silencers has revealed that these CREs are cell line-specific and may function as enhancers or silencers depending on the cellular context [31,32,33,34]. Most MRRs are unique to individual cell lines and may overlap with super-enhancers in other cell types [32]. For example, a MRR near the *CPED1* gene was identified in GM12878 cells, but the same genomic locus was identified as a super-enhancer in K562 cells, indicating the importance of silencers in the precise control of gene transcription in different cells [32]. Pang and Snyder [34] treated K562 cells with phorbol 12-myeistate 12-acetate (PMA) to induce megakaryocytic differentiation, and identified 1,245 silencers that were different between the differentiated cells and the wild-type K562. This suggests that the repressive role of silencers is tissue-specific and necessary for cell differentiation. The cell type specificity of human silencers was also reported by Huang et al. [31] and Doni Jayavelu et al. [33]. Such uniqueness and specificity of human silencers suggests they might be primed for specific gene regulation in different cellular environments and different developmental stages.

Silencers have also been suggested to function in drug resistance. Deletion of silencer regions linked to the drug transporter genes *ABCC2* and *ABCG2* increased chemo-resistance to doxorubicin, daunorubicin, and etoposide [34], suggesting that genetic variation in silencer regions may impact both drug delivery and personalized medicine. Apart from roles in development, differentiation, and drug resistance, silencers may also play roles in various diseases such as cancer.

## 10. Silencer and Repressor Dysregulation in Cancer

Altered expression of PcG proteins has been commonly observed in human cancers. EZH2, the enzymatic subunit of PRC2 [103], is recurrently mutated and highly expressed in numerous cancers [104]. EZH2 silences genes coding for transcription factors and cell-cycle regulators in metastatic hormone-refractory prostate cancer, and EZH2 overexpression was associated with worse disease progression [105]. Similar findings correlating high levels of EZH2 with aggressiveness and advanced disease have emerged in other human cancers [104]. Besides EZH2 overexpression, various point mutations in EZH2 have also been reported to result in high levels of H3K27me3 signals, including mutations at tyrosine 641 (Y641) [106,107], alanine 677, and alanine 687 (A677 and A687) [108,109], thereby inhibiting tumor suppressor genes to favor cancer progression.

Besides EZH2, overexpression of other PcG proteins such as SUZ12 and BMI1 have also been shown to favor cancer progression. In epithelial ovarian cancer, high expression of *SUZ12* inhibited cell apoptosis by inhibiting pro-apoptotic genes such as harakiri (*HRK*) [110]. In non-small cell lung cancer, SUZ12 promotes cell proliferation and metastasis by decreasing E2F transcription factor 1 (*E2F1*) gene expression [111]. Overexpression of *BMI1* occurs in both solid tumors and hematological cancers and is linked to proliferation, invasion, and poor patient survival [112]. BMI1 is also implicated in the self-renewal of cancer stem cells [113,114]. Together, these results suggest that aberrant Polycomb repression plays an important role in cancer.

Apart from curbing the expression of neuronal-specific genes in non-neuronal tissues, REST also functions as a tumor suppressor gene [115]. Loss of REST function in colon, lung, breast, and prostate cancers increased expression of genes involved in cell proliferation and survival [116]. In particular, reduced REST function activated neuroendocrine genes, leading to an aggressive neuroendocrine carcinoma-like phenotype [116,117].

Taken together, silencers play important roles such as cell development, differentiation, and perhaps even evolution. Several lines of evidence suggest that alteration of silencer sequence or the silencer-associated repressor can lead to various diseases, including cancers.

## 11. Conclusions and Future Directions

Spatial and temporal control of gene expression is crucial for multicellular organism development, and dysregulation of these regulatory mechanisms can lead to various diseases such as cancer [118,119]. CREs are thought to control precise gene expression patterns. Activating CREs such as enhancers and super-enhancers have been extensively studied [120,121], while repressive CREs such as silencers are less well understood.

Human silencers have been shown to exist to play gene repression roles, and multiple human silencer examples including proximal silencers and looping silencers have been elucidated in different cellular contexts. Although multiple experimental and bioinformatics methods have been proposed for systematic genome-wide identification of human silencers, [31,32,33,34], these works are still at the nascent stage, and the scientific community has not arrived at a consensus on the definition and identification of silencers. Further work needs to be done to improve on these current methods and to formulate novel methods, in order to identify a gold standard for the genome-wide identification of human silencers.

Different subclasses of silencers may be endowed with subclass-specific chromatin signatures or repressor binding profiles. In different cellular contexts and different developmental stages, the chromatin signatures and repressor binding profiles of these elements can switch between repressive and activating modes, functioning as both silencers and enhancers [32,33,122], allowing for lineage-specific enhancer/silencer function and precise gene expression patterns. As we improve on silencer identification methods, it is imperative that we determine the mechanism and cellular context in which these elements operate as silencers. The ENCODE consortium has generated an encyclopedia of candidate human CREs using histone modification and chromatin accessibility signatures, identifying potential promoter and enhancers across different cell types [123]. We look forward to an expansion of this registry to encompass candidate silencer elements, by applying a similar approach with repressive histone modifications such as H3K27me3 and H3K9me3. This will allow us to better dissect the active and repressive functions of CREs in different cellular contexts and lineages.

There are heated debates on whether super-enhancers are merely an assembly of nearby enhancers or whether the constituent enhancers work cooperatively to regulate the same gene [124,125]. Enhancers have been shown to function in different modes, including hierarchically [126], additively [127], or redundantly [128]. Similar questions need to be asked about silencers as well, including whether silencers function additively or synergistically to silencer gene expression. In particular, do silencer clusters, such as MRRs, have greater repressive function, similar to super-enhancers?

Super-enhancers are enriched with various activators such as BRD4, and have been shown to control cell identity [120]. In cancer, aberrant acquisition of super-enhancers can drive oncogene expression, and mutations of super-enhancers have been found in multiple cancer cell types [129,130]. Given the prevalence of super-enhancer dysregulation in cancer, inhibition of super-enhancer-driven gene expression has become a popular therapeutic strategy, with drugs targeting BRD4 and CDK7 [131,132,133] being developed for anticancer therapies.

Similar to super-enhancers, the importance of silencers towards cell identity has been demonstrated, and perturbation of silencers has been shown to lead to changes in cell identity [32]. Genome-wide association studies (GWAS) have demonstrated that disease-associated single-nucleotide polymorphisms (SNPs) are enriched at silencer regions [31,33,134], indicating that mutations at silencers can have phenotypic consequences. However, further experimental validation using knock-out or point mutation studies are needed to connect disease-specific silencer mutations to their target genes. Targeting dysregulated silencer function in cancer by inhibiting repressors and ncRNAs or perturbing chromatin interactions and phase separated condensates may be an important novel therapeutic strategy against cancer. We propose three major research directions to focus on: 1. Explore the factors and mechanisms that control silencer function; 2. Identify disease-specific silencer dysregulation by comparing healthy people and cancer patients; 3. Screen for drugs that perturb silencer function.
